# A concise review of carbon nanotube's toxicology

**DOI:** 10.3402/nano.v4i0.21521

**Published:** 2013-12-03

**Authors:** Seyed Yazdan Madani, Abraham Mandel, Alexander M. Seifalian

**Affiliations:** 1 UCL Centre for Nanotechnology & Regenerative Medicine, UCL Division of Surgery & Interventional Science, University College London, London, UK; 2 Royal Free London NHS Foundation Trust Hospital, London, UK

**Keywords:** carbon nanotubes, toxicology, distribution, administration

## Abstract

Carbon nanotubes can be either single-walled or multi-walled, each of which is known to have a different electron arrangement and as a result have different properties. However, the shared unique properties of both types of carbon nanotubes (CNT) allow for their potential use in various biomedical devices and therapies. Some of the most common properties of these materials include the ability to absorb near-infra-red light and generate heat, the ability to deliver drugs in a cellular environment, their light weight, and chemical stability. These properties have encouraged scientists to further investigate CNTs as a tool for thermal treatment of cancer and drug delivery agents. Various promising data have so far been obtained about the usage of CNTs for cancer treatment; however, toxicity of pure CNTs represents a major challenge for clinical application. Various techniques both *in vivo* and in *in vitro* have been conducted by a number of different research groups to establish the factors which have a direct effect on CNT-mediated cytotoxicity. The main analysis techniques include using Alamar blue, MTT, and Trypan blue assays. Successful interpretation of these results is difficult because the CNTs can significantly disrupt the emission of the certain particles, which these assays detect. In contrast, *in vivo* studies allow for the measurement of toxicity and pathology caused by CNTs on an organismal level. Despite the drawbacks of *in vitro* studies, they have been invaluable in identifying important toxicity factors, such as size, shape, purity, and functionalisation, the latter of which can attenuate CNT toxicity.

The 21st century has seen major advances in nanotechnology, which can be applied to a wide array of scientific disciplines, including medicine. This gives rise to the field of nanomedicine, which holds great promise in providing novel methods of diagnosing and treating many debilitating diseases, such as atherosclerosis, cancer, multiple sclerosis and many more (1). Despite the recent advances in potential clinical applications of nanoparticles, their safe use in humans is a growing concern (2). Currently, our understanding of nanoparticle toxicity, or nanotoxicity, is limited due to their minimal size. Also, there are few data available on the long-term impact of nanoparticles on human health.

There are different ways in which a nanoparticle can enter the human body, including penetration through the skin, ingestion, inhalation, and injection (1). Carbon nanotubes (CNTs) offer a wide range of applications due to their unique atomic configuration, mechanical, optical, and electronic properties, high surface-area-to-volume ratios and facile functionalisation. Any use of CNTs in humans, be it for diagnostic or treatment purposes, would involve considerable exposure to CNT particles and therefore understanding their safe use is of paramount importance (3). There has been a substantial effort in recent years to determine the nature of nanotoxicity in living organisms and cells. To that end, scientists have examined CNT toxicity in several cell lines (4, 5). Their findings suggest that various factors including functionalisation, purity, size, length, diameter, surface chemistry can determine CNT toxicity (6). As well as using *in vitro* studies to explore the effect of various factors on CNT toxicity, *in vivo* studies have also played a major role for investigating the CNT toxicity. *In vivo* studies are favoured over *in vitro* studies, since they allow for nanotoxicity to be measured on an entire organism, rather than in cell culture. Recently *in vivo* toxicology studies conducted with a focus on multiple organ systems have suggested that CNTs initiate a toxic response within these multiple organ systems. In this review, we discuss the factors that determine CNT toxicity and the methodologies currently used in toxicology studies.

## Structure of CNTs

Carbonic structures come in diverse shapes and configurations both within compounds and as elementary substances. Various carbonic allotropes include charcoal, graphite, carbon black, diamond, and CNTs. Graphite, which is made from several layers of single-atom width, planar sheets of hexagonal bonded carbon in a honeycomb crystal lattice is known as graphene when present as single sheets and takes on novel properties. Furthermore, single or multiple graphene sheets can be folded into cylindrical structures to give single- and multiple-walled CNTs, respectively, which again take on unique and novel properties (5, 7, 8).

**Fig. 1:**
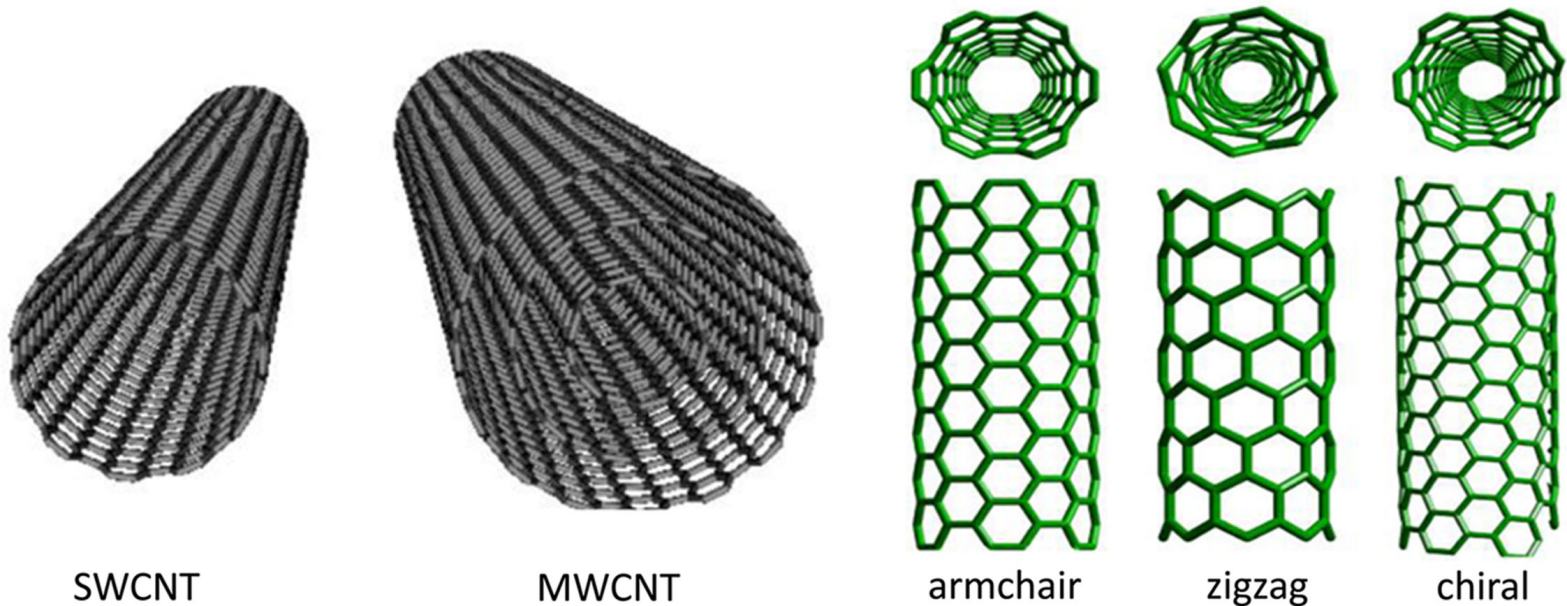
Different types of CNT in grey are models of either single-walled or multi-walled CNTs and in green are the various forms of SWCNTs.

CNTs are hollow graphitic nanomaterials with precise structural arrangements and order throughout giving them a range of properties such as ultra-light weight, high surface area and a high aspect ratio (9). In general, CNTs are separated into two classifications; single-walled (SWCNTs) and multi-walled (MWCNTs). As the names implies, SWCNTs are mono-cylindrical carbon layers with a diameter range of 0.4–2 nm, where a higher synthesis temperature produces SWCNTs with a greater diameter than a lower synthesis temperature. However, MWCNTs consist of a number of cylindrical carbon sheets with an average diameter of 1–3 nm for the central cylindrical tubes and 2–100 nm for the external cylindrical tubes. MWCNTs structures can be split into two categories based on their arrangements of graphite layers: one has a parchment-like structure which consists of a graphene sheet rolled up around itself and the other is known as the ‘Russian doll’ model where layers of graphene sheets are arranged within a concentric structure. Conversely, SWCNTs’ structures are organised in harmony with chiral, armchair, helical, and zigzag arrangements (Fig. 1).

## Methods of production

One method to synthesise CNTs is by heating graphite and carbon black in a controlled flame atmosphere. However, this method gives rise to irregularities in size, shape, quality, purity, and the mechanical strength of the CNT. In response, catalytic decomposition of hydrocarbons and laser ablation techniques has been suggested as an alternative method, without the above-mentioned issues. Indeed, by using different synthesis methods, CNTs can be synthesised with different unique properties, which relate to their intended function (2).

Because of the unidimensional structure of SWCNTs and their high surface area which increases drug-loading capacity, SWCNTs are more efficient in drug delivery than MWCNTs. Previous studies have shown that the blood circulation time for an anticancer drug agent conjugated with a SWCNT is much longer compared to the anticancer drug on its own. This can then result in the tumour cells having more sustained and prolonged uptake of the drug particles via enhanced retention and permeability effects.

## How can CNTs enter the body?

Methods of CNT administration include: oral, intravenous (IV) injection, inhalation, transdermal, subcutaneous injection, and intraperitoneal. Although the former four methods are the most common for human use, the majority of *in vivo* data collected to date are based on intratracheal instillation and inhalation in rodents and there have been only a small number of experiments in relation to oral and IV routes which are more relevant to humans in regard to nanomedicine. Although there is no clear indication of the most toxic method of entry of the CNT into the body as yet, some studies have indicated that IV injection, oral, and dermal administration of CNTs would only result in a mild inflammation, whilst inhalation of CNTs may result in severe inflammation (2). After nanostructures are introduced into the body, they initially interact with biological entities such as proteins or cells and can either retain their original structure or be metabolised. Passive diffusion and energy-dependent endocytosis are the two methods suggested for CNT entry into living cells (4). They can also be distributed to various parts of the body, from where they can either remain, translocate, or be excreted. However, the time frame of these processes is largely unknown (10).

## Can CNTs damage the body's organs?

**Fig. 2:**
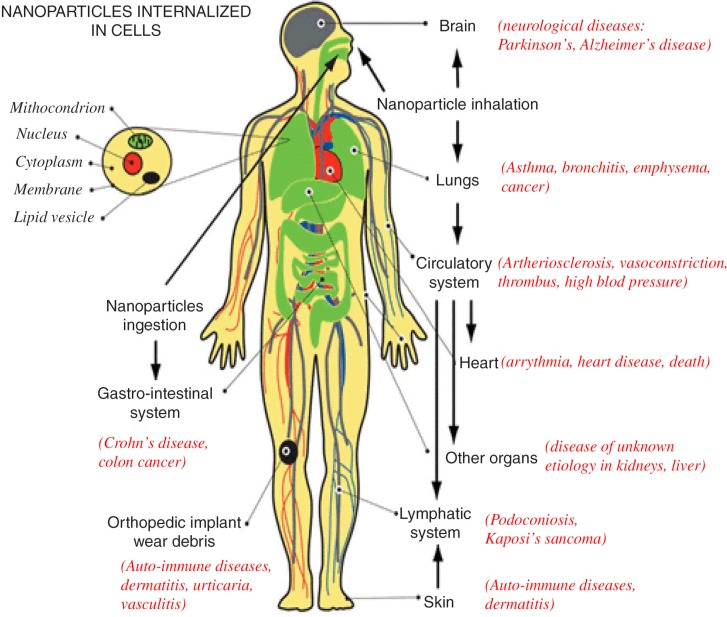
Effect of CNT on body's organs. Illustrates the disease that can be developed as a result of the exposure of nanoparticles to different parts of human organs (11).

In recent years, as the use of CNTs in industry has increased, so has their exposure to humans. As a result, different research groups have been examining the effect of these materials on human health (5). Their effect appears to be related to their method of administration. Various research groups have found that exposure of nanoparticles to the respiratory system could result in asthma, bronchitis, emphysema, and lung cancer. Entry of nanoparticles through the gastrointestinal tract (GIT) could lead to Crohn's disease and colon cancer. Furthermore, it has been discovered that the nanoparticles exposure to the circulatory system may result in blood clotting and heart disease (11) (Fig. 2).

It is important to note that not all nanoparticles are toxic. As alluded to earlier, altering the shape, size, and composition can modify the nanotoxicity of CNTs. However, the similarity of the needle-like fibre shape of CNTs to asbestos particles and has prompted researchers to investigate nanotoxicity of CNTs on the respiratory system further (12). Asbestos fibres are long, thin crystals, which due to their light weight can be inhaled into the lungs and cause pathogenicity upon their inhalation (13). As mentioned previously, one method of CNT's entry into the body is through the respiratory system. Different research groups have conducted various *in vivo* experiments to investigate the toxicity on the respiratory system following CNT exposure.

## Intratracheal instillation

One method to investigate the toxicity of the CNT on the respiratory system is to inject the CNT through the trachea. This is known as an intratracheal instillation technique. Whilst the majority of studies using intratracheal instillation of CNTs have indicated severe toxicity to the respiratory system, studies involving direct inhalation of CNTs indicated no major damage to the respiratory organs (12, 14). In an experiment, a group of mice were intratracheally instilled with 0, 0.1, or 0.5 mg of SWCNT, carbon black, or quartz. The mice were then euthanised 7 and 90 days post-instillation for histopathological study. The results showed the presence of epithelioid granuloma in all SWCNT groups post-7 days of intratracheal instillation of SWCNT and interstitial inflammation in some cases. In all SWCNT groups post-90 days, the results showed a significant inflammation. However, peribronchial inflammation and necrosis that had extended into alveolar septa were observed in some of the cases. However, the lungs of mice which were exposed to carbon black appeared normal at all doses on both 7 and 90 days post-injection, and a mild inflammation in the case of all doses of quartz was observed (13). Various experiments have also been conducted on MWCNTs injected intratracheally and the results have also indicated a severe toxicity. In one experiment, MWCNTs (0.5, 2, or 5 mg per rat) were introduced into rat tracheas, resulting in inflammatory and fibrotic reactions at all doses (15). In another experiment, 0.5 ml of 500 µg/ml of MWCNT or crocidolite was administrated five times over 9 days into the lungs of F344 rats through the trachea. The pleural cavity lavage fluid, lung, and chest wall were then collected. The results show the presence of MWCNT and crocidolite in aleveolar macrophages and mediastinal lymph nodes. However, it was found that only administration of MWCNT will result in mesothelial proliferation (16). Interestingly, the toxicity of both SWCNT and MWCNT following their intratracheal injection is dose dependent. This has also been demonstrated following intraperitoneal injection of CNTs. In an experiment, three groups of mice were given a single intraperitoneal injection of 300, 30, and 3 µg and monitored over 1 year. The mesothelioma incidence was 19/20, 17/20, and 5/20, respectively. The results also showed dose dependence in peritoneal adhesion and granuloma formation (17).

## Inhalation

As mentioned above, as well as using intratracheal instillation of the CNT into the lung, the inhalation method has also been used to investigate the CNT toxicity on the respiratory system. In one study, C57BL/6 adult mice were exposed by whole-body inhalation to 0.3, 1, or 5 mg/m^3^ of MWCNT for 7 or 14 days (6 h/day) compared to air as a control. The result did not show significant lung inflammation or tissue damage at any of the doses (18).

## Dermal and subcutaneous

**Fig. 3:**
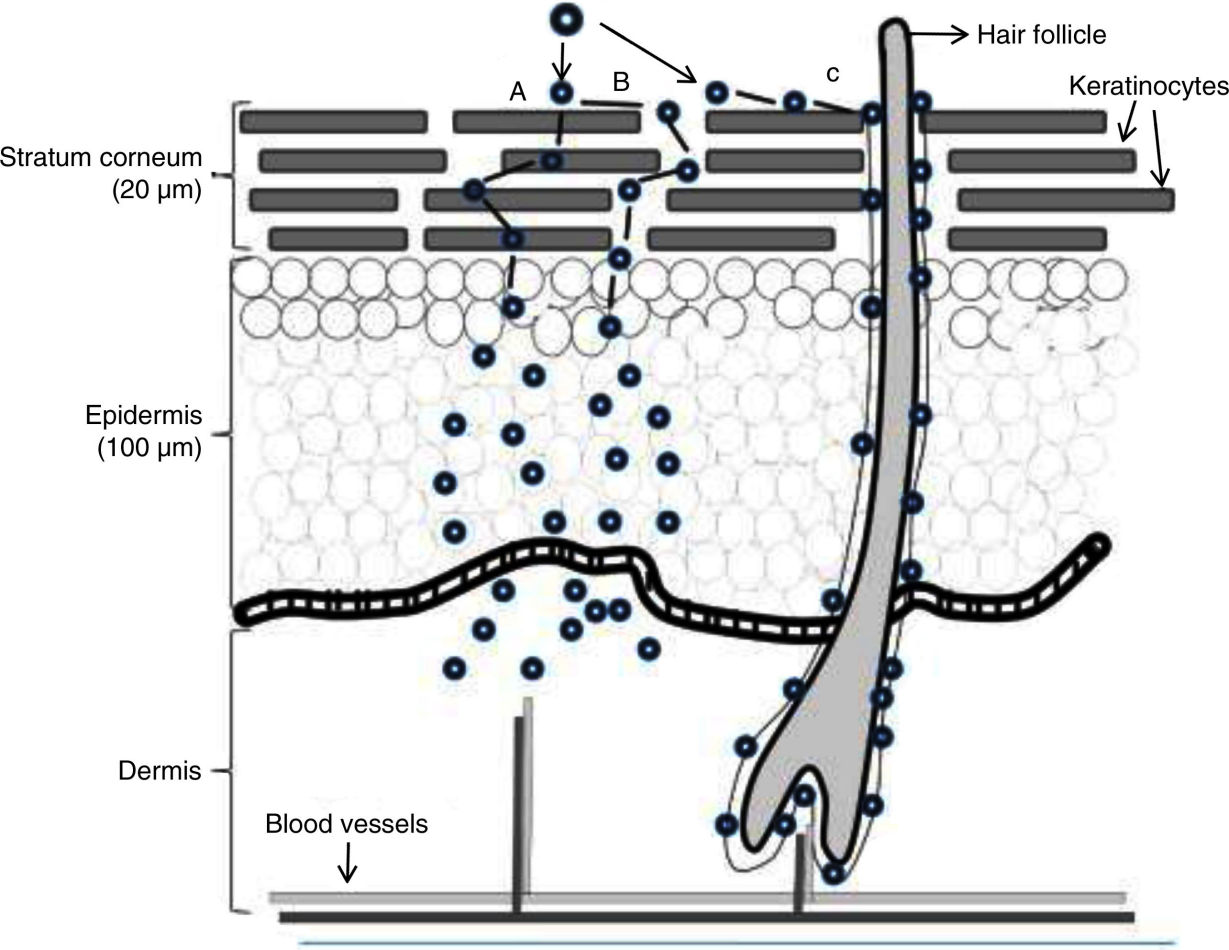
Potential routes for nanoparticle delivery through the skin. This occurs either via the (A) intercellular, (B) transcellular or (C) transappendageal pathway. The nanoparticles then pass through the epidermis and dermis before reaching the blood vessels. Reproduced from Valenzuela and Simon, 2012 (21).

Whilst the similarity between CNTs and asbestos has prompted research into the effects of CNTs on the respiratory system, CNTs can enter the body through other routes, which may be toxic in other ways. Various *in vivo* experiments have been conducted to investigate the effect of CNT on dermal contact. According to one study, exposure of non-purified CNTs to mice skin caused oxidative stress, depletion of glutathione, an increase of dermal cell number, and skin thickening. The results showed that purity of the CNT is an important determinant of its toxicity when administered dermally (11, 19). Metals, particularly iron, are one type of CNT impurity. These materials are found in the CNT's structure as they are used during the synthesis process. In an experiment, mouse skin was exposed to either pristine CNT or functionalised CNT, and the results showed hair loss only in the case of pristine CNTs as opposed to the functionalised CNTs. This was due to the significant removal of iron from the CNT following the functionalisation. Although works from various research groups have produced great amounts of information on the toxicity of CNTs on skin, there is limited information available on CNTs mechanism of toxicity and skin absorption. It has been suggested that the CNT mechanism of absorption differs from other chemicals due to the different sizes and engineering design. Upon CNT exposure to the skin, the CNTs first have to penetrate the stratum corneum layer, the outermost layer of the epidermis that consists of several sheets of keratinised dead cells. They then need to travel through the viable epidermal layers to gain access to the capillaries within the papillary layers of the dermis to get into the systemic circulation (20). Generally, it has been suggested that upon the exposure of a nanoparticle to the skin's surface, these materials can be absorbed by different methods. These include travelling between the stratum corneum cells, through the cells (transcellular) or through the hair follicle or sweat ducts (transappendageal) (21) (Fig. 3). Although absorption routes may differ between CNTs and other chemicals, they may also differ between different types of CNTs, due to differences in size, shape, surface modifications, and composition (1, 20).

**Fig. 4:**
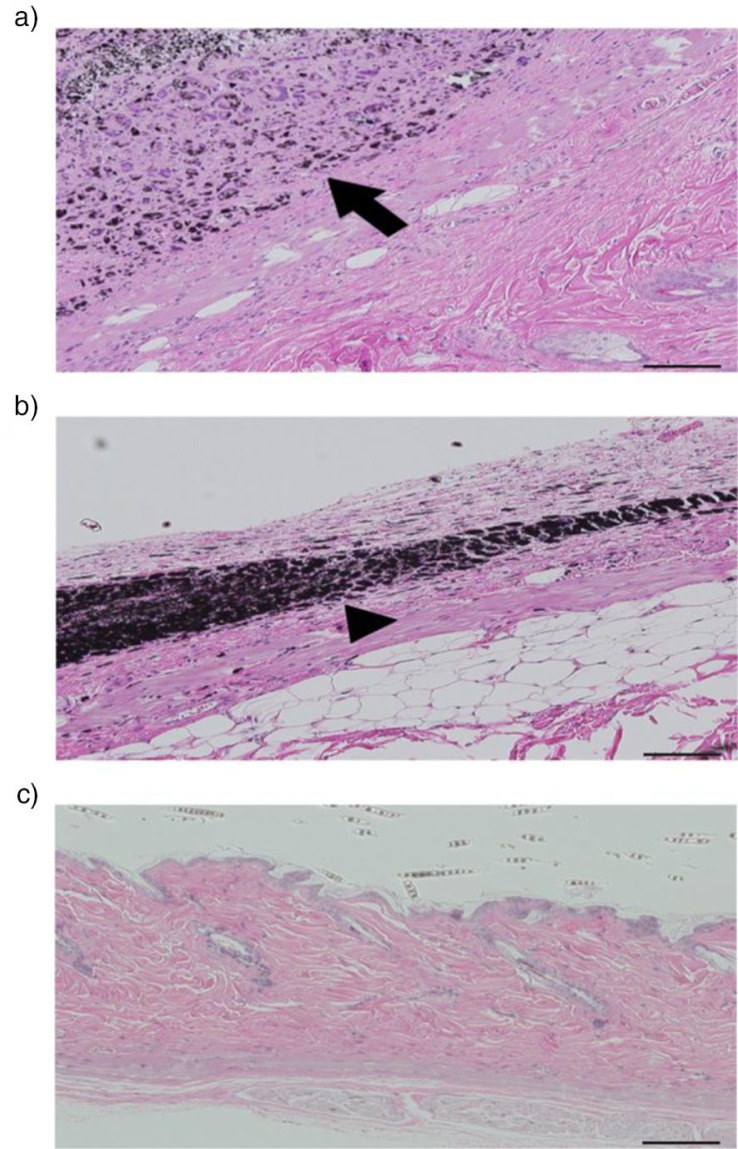
Skin behaviour of mice following its exposure to different compounds. Illustrates the skin of rasH2 mice and the way in which CNT, carbon black, or MNU will behave 26 weeks after the injection. CNT is illustrated by arrow and the arrowhead is showing the carbon black, scale bar: 100 mm. a) CNT, no inflammatory cells such as neutrophils and lymphocytes it observed on the surrounding tissue of the injection site. b) Carbon black, no neoplasm was observed. Phagocytosis process can be observed. c) Presence of squamous tumour cell was detected at the injection site of mice (22).

Subcutaneous injection of CNT is another method of administrating the CNT into the body. In an experiment conducted by a group of researchers in Japan, MWCNT, carbon black, and *N*-methyl-*N*-nitrosurea (MNU) were subcutaneously injected into mice. The histological results indicated minimum carcinogenicity of MWCNT in comparison to the other materials. In addition, no neoplasms were detected at the injection site and the inflammatory cells such as neutrophils and lymphocytes were not observed on surrounding tissues in the case of MWCNT injection (22) (Fig. 4).

## Intraperitoneal

As well as intratracheal and dermal entry routes, CNTs can be injected intraperitoneally. In one experiment, female imprinting control regions (ICR) mice were administered intraperitoneally (2 mg/kg body weight) with either MWCNT, carbon black, or crocidolite (blue asbestos) or a control (2% sodium carboxymethyl cellulose). MWCNT administration resulted in the liver becoming round in shape, the presence of fibrous adhesion on internal organs and deposits on the surface of the liver and diaphragm. In addition, the results showed an overexpression of mRNA for genes of T helper 2 cytokines, T helper 17 cytokine, and pro-inflammatory cytokines/chemokines in peritoneal cells 2 weeks after MWCNT peritoneal injection. An increase in the number of leukocytes, monocytes, and granulocytes in the peripheral blood within 1 week of MWCNT peritoneal injection was also observed in addition to an increase in IgM and IgG levels. No changes were recorded following the addition of carbon black or crocidolite administration (23).

Whilst the studies above provide an insight into how the route of entry of a CNT can determine the type of toxicity, it is important to be able to compare these different routes of entry in a standardised manner. As a result, different research groups have also used these methods for investigating the difference between intratracheal and intraperitoneal administration on possible teratogenicity of MWCNT. In one experiment, MWCNTs were administered intraperitoneally or intratracheally to pregnant mice on day 9 of gestation, all foetuses were removed from the uterus on day 18 of gestation and examined for external and skeletal abnormalities. The results indicated the presence of severe foetal abnormities in the case of intraperitoneal administration of all MWCNT-treated group (2, 3, 4, and 5 mg/kg body weight). However, in the case of intratracheal administration of MWCNT, foetal abnormalities were only observed at 4 and 5 mg/kg body weight and no abnormalities were indicated at 3 mg/kg (24).

## Factors affecting the toxicity of the CNT

**Fig. 5:**
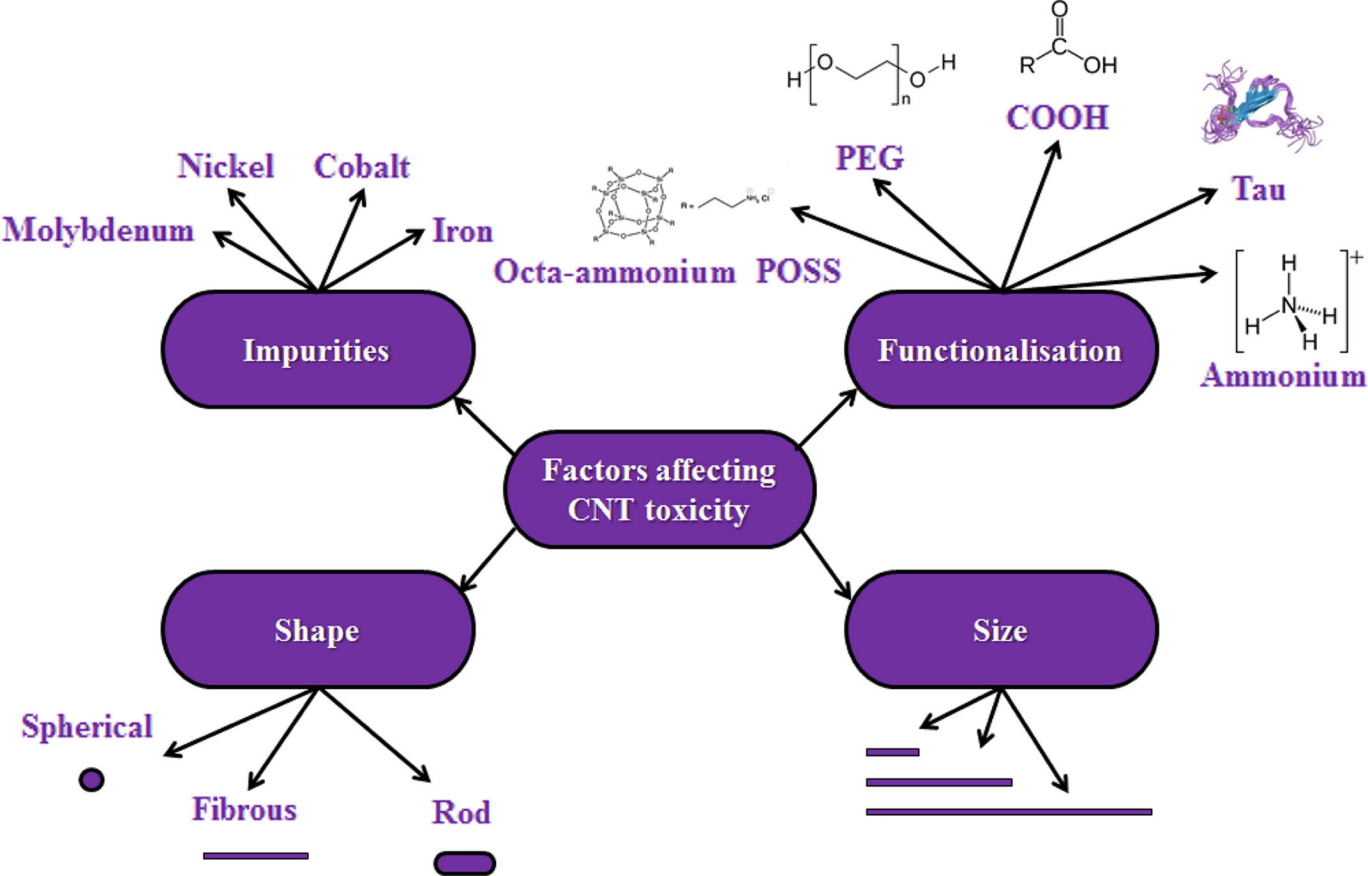
Summary of the factors affecting CNT toxicity. The figure illustrates with examples the many factors that have been shown to mediate CNT toxicity, including impurities, functionalisation, shape, and size. Although there is no consensus on the exact effect of all of these factors on CNT toxicity, building a picture of such factors will assist future design of safer CNTs.

The toxicity of CNTs can be affected by a wide range of factors (Fig. 5 and Table 1).

**Table 1:** List of some of the functional groups, which have used by different groups for reducing the CNT's toxicity

Year	Type of CNT	Functional group	Type of conjugation	*In vitro/in vivo*	Outcome
2012 (4)	SWCNT	COOH	NA	Human intestinal cell line	Reduce cell's toxicity in comparison to pristine SWCNT
2012 (25)	SWCNT	PEG	Non-covalent	Mice model	Granulomas and fibrosis detected as a result of the pure MWCNT, no toxicity detected post-functionalisation
2012 (26)	MWCNT	DEX	Reacting DEX with MWCNT functionalised with –COOH group	Lung epithelial cancer cell line (A-549)	DEX-functionalised MWCNT resulted in more cell death than DOX only
2012 (3)	MWCNT	PEG	Covalent	Human breast cancer cell line (SKBR3)	Both short-length MWCNT and short-length MWCNT-PEG determined no toxicity on the cell line
2012 (27)	MWCNT	CSH	NA	Fibroblast cells (L929)	No significant differences in cell proliferation between the experimental and control groups
2012 (28)	MWCNT	Arginine and lysine	Microwave radiation	Gram-negative bacteria	Post-functionalisation, antibacterial activity was increased
2012 (29)	SWCNT	COOH	Covalent	RAW264.7	Similar toxicity between pristine and functionalised SWCNT was observed
2011 (30)	MWCNT	COOH	Covalent	HMMs	Twenty microgram per millilitre MWCNTs cell viability was reduced by 13.3%. MWNT-COOH was not significantly toxic
2011 (31)	SWCNT	PEG	NA	Neuronal PC12 cells	SWCNTs elicited cytotoxicity in a concentration-dependent manner. SWCNT–PEGs exhibited less cytotoxic potency.
2010 (32)	SWCNT	Range of natural (gum arabic, amylose) and synthetic	Non-covalent	Rat liver epithelia cells WB-F344	With all functional group except Triton X-100, no inhibition of GJIC observed.
2010 (33)	MWCNT	PAMAM	Covalent	Human osteosarcoma MG-63 cells	No obvious cytotoxicity at concentration <25 microgml^−1^ over 24 h
2009 (34)	MWCNT	PEI	Covalent bonding to acid-treated MWCNT	Human thyroid cancer cell line	Surface charge of the functional group plays an important role in cytotoxicity
2003 (8)	SWCNT	Epoxy	Mixing the functionalised SWCNT with Epoxy	NA	Considerable improvement in the dispersion
2000 (35)	MWCNT	4-HN	NA	Embryonic rat-brain neurons	Pristine MWCNT: neurons extend only one or two neuritis. functionalised MWCNT: multiple neuritis

DEX: dexamethasone, DOX: doxycycline, CSH: calcium sulfate hemihydrates, COOH: carboxylic acid, PAMAM: polyamidoamine, PEI: polyethyleneimine, 4-HNE: 4-hydroxynonenal, PEG: polyethylene glycol.

### Functionalisation

**Fig. 6:**
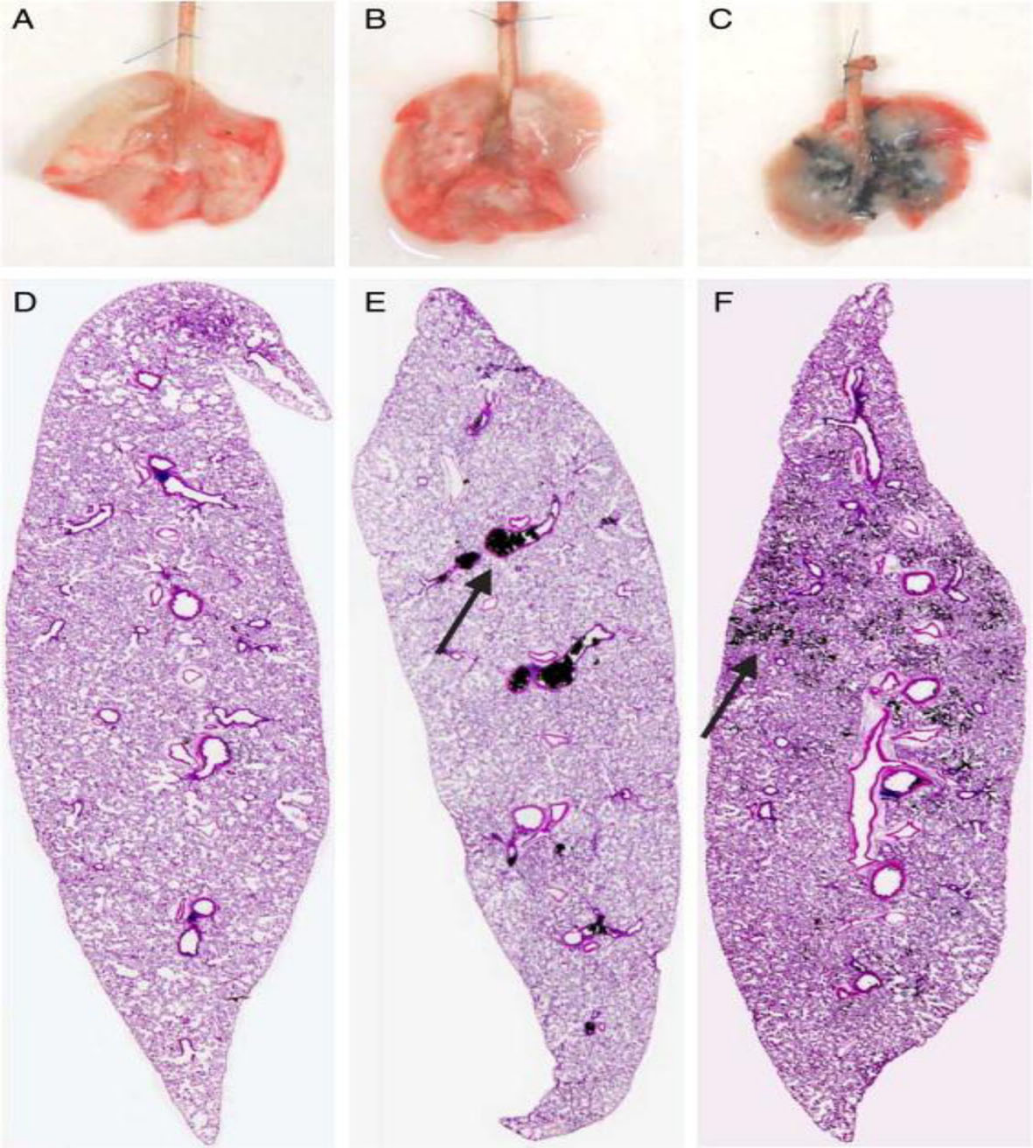
Distribution of CNT following the intratrachial administration. Illustrate the dispersion of saline (A, D), CNT (B, E) and grinded CNT (C, F) in the lung of rat following the intratrachial administration. As it can be clearly illustrated in case of the grinded CNT, the CNT is uniformly dispersed in the lung in compression to the CNT (36).

Various studies including our own (unpublished data) have shown that the exposure of pristine CNT significantly reduces the proliferation rate of cells and induces cell cycle arrest, apoptosis, and necrosis (2). In general, it has been observed in the biological milieu that the CNT will interact with proteins and interfere with their structure, possibly causing cell death. For this reason, it has been suggested that efficient surface coating of CNTs is a crucial stage before using the CNTs in a biological environment (1). Functionalisation is the process whereby CNTs, which normally aggregate into clusters, are separated and coated with certain molecules. It has been established by various research groups that functionalisation can significantly improve the dispersibility and biocompatibility, whilst reducing the toxicity of CNTs. One study, which measured the dispersion of agglomerated and ground MWCNT following intratracheal administration into rat lungs, found that the ground MWCNT was significantly more dispersed in comparison to the agglomerated MWCNT (17) (Fig. 6). Results from other studies have suggested that functionalisation will result in the complete disappearance of the CNTs’ toxicity (37). Whilst there are many ways to functionalise CNTs, one of the most common approaches is to coat the CNT surface with polyethylene glycol (PEG). PEG is a well-known, widely used, biocompatible polymer, which can reduce CNT toxicity *in vivo*, improve pharmacokinetic behaviour, prolong the blood circulation half-life, and reduce the reticuloendothelial system (RES) capture (38). In terms of the bonding, these materials can coat the CNT via covalent or non-covalent bonding. Studies have shown that covalent bonding of PEG to the surface of the CNT, using increased molecular-weight PEG and more branched PEG can further improve the CNT pharmacokinetic behaviour and decrease its toxicity (39). One report indicated that using PEG2000 results in a CNT blood circulation half-life of 1.2 h and that using PEG5000 could increase it to 5 h. Another report found that using branched PEG could improve the blood circulation to as long as 15 h (40). In terms of the excretion, the results also demonstrate that pristine CNT is minimally excreted from the body, whilst PEG-coated CNT would be excreted through urine and faeces. In terms of the toxicity, some of the studies have demonstrated a complete disappearance of the CNTs toxicity following its conjugation to the PEG. CNT-PEG's toxicity has only been reported at high doses of CNT (40). In one study, the group injected SWCNT–PEG (3 mg/kg) intravenously into mice. Their monthly observation of blood count and serum chemistry as well as careful necropsy and tissue histology examinations 4 months post-injection, indicated no change in any of the parameters. This observation suggests that functionalisation of SWCNT with PEG can be made safe for *in vivo* biological application (37, 38). In another study, it was found that by using an appropriate functionalisation technique, CNT toxicity could be removed even at very high doses. In this study, a large dose of ammonium-functionalised CNT (20 mg/kg, was injected into mice. After 24 h, the mice showed no physiological and pathological changes (7, 41). CNTs can also be functionalised with Tau (42). One study demonstrated that Tau-coated CNTs resulted in very low cell inflammation and mitochondrial destruction at very high CNT dose. The study also showed that after mice were injected with Tau–CNT, no changes in serum biochemical index or histopathological characteristics were observed, in comparison to pristine CNT (40). Functionalisation with carboxylic acid (–COOH) is another method of reducing CNT toxicity. In an unpublished experiment carried out here at The Centre for Nanotechnology and Regenerative Medicine, both pristine and functionalised MWCNT and SWCNT with carboxylic acid at various concentrations (0.125, 0.250, 0.500, and 1.000 mg/ml) were added to colorectal cancer cells (HT29). Cell viability and metabolism were observed over 24, 48, and 72 h. Results clearly illustrated that in both cases of MWCNT and SWCNT, functionalisation resulted in significantly higher cell viability and metabolism in comparison to pristine SWCNT and MWCNT. In another experiment, the effect of the functionalisation period was also investigated. In this experiment, both types of CNT were treated with HNO_3_ and H_2_SO_4_ for 0.5, 2, and 5 h. Results showed that in the case of both MWCNT and SWCNT, the longer the functionalisation period, the greater the number of viable cells. In addition to functionalisation of CNT with –COOH, we have also investigated the effect of coating the surface of CNTs with OctaAmmonium POSS. In this experiment, the OctaAmmonium POSS was conjugated to the CNT's surface through covalent and non-covalent bonding. In the case of non-covalent bonding following ultra-sonication, the OctaAmmonium POSS was added to the CNTs and stirred for a period of 2 h. For covalent bonding, the CNTs were initially treated with HNO_3_ and H_2_SO_4_ to obtain carboxylic acid group on their surface. The OctaAmmonium POSS was then reacted with NaOH to convert the ammonium group of the OctaAmmonium POSS into amine groups. Following the presence of amine group on the surface of the OctaAmmonium POSS, it was reacted with the CNTs in the presence of linker agents. The OctaAmmonium POSS attached covalently or non-covalently to the CNTs’ surface and were both added to a breast cancer cell line in different experiments. The result clearly illustrated that in both SWCNTs and MWCNTs, the covalent attachment of Octa-Ammonium-POSS to the CNT's surface resulted in lower cell death. This is due to the fact that the OctaAmmonium POSS acts as an extra coating or barrier on the CNT surface in addition to the –COOH group, even though –COOH alone has been shown to reduce CNT toxicity. In one study, Caco-2 cells (human intestinal cell line) were treated with –COOH-functionalised SWCNTs (COOH–SWCNT). The results indicated a significant reduction in cell viability and morphological change following COOH–SWCNT treatment. Their study focused on the mechanism of COOH–SWCNT-mediated cell death. They concluded that oxidative stress is triggered by the addition of COOH–SWCNT group (4). In another study, the lungs of C57BL/6 mice were exposed to COOH–SWCNTs. Histopathological examination revealed that COOH–SWCNT may induce acute lung injury in mice via pro-inflammatory cytokine storm signalling through the NF-κB pathway (43).

### Purity

Co, Fe, Ni, and Mo are the most common materials which are widely used in CNT synthesis. These metals are used as a catalyst to promote the CNT's growth process during synthesis (44). Following synthesis, residual metal is normally encapsulated in a layer of carbon, either amorphous soot or layers of graphite. It has been discovered that metal impurities is one of the main factors that determines CNT toxicity, resulting in cell death through various mechanisms including mitochondrial destruction and oxidative stress (4). The metal catalyst content should always be taken into account when the CNT's toxicity is investigated (37). As a result, CNT purification is one method used to reduce CNT cytotoxicity. Functionalisation is a way of improving CNT purity (10). One way of doing so is through ultra-sonication. One study demonstrated that ultra-sonication of CNTs for as short as 5 min can significantly reduce their toxicity. It has been suggested that one reason why ultra-sonication has this effect is because ultra-sonication promotes the release of a metallic impurities into solution (45).

### Shape of nanoparticles

**Fig. 7:**
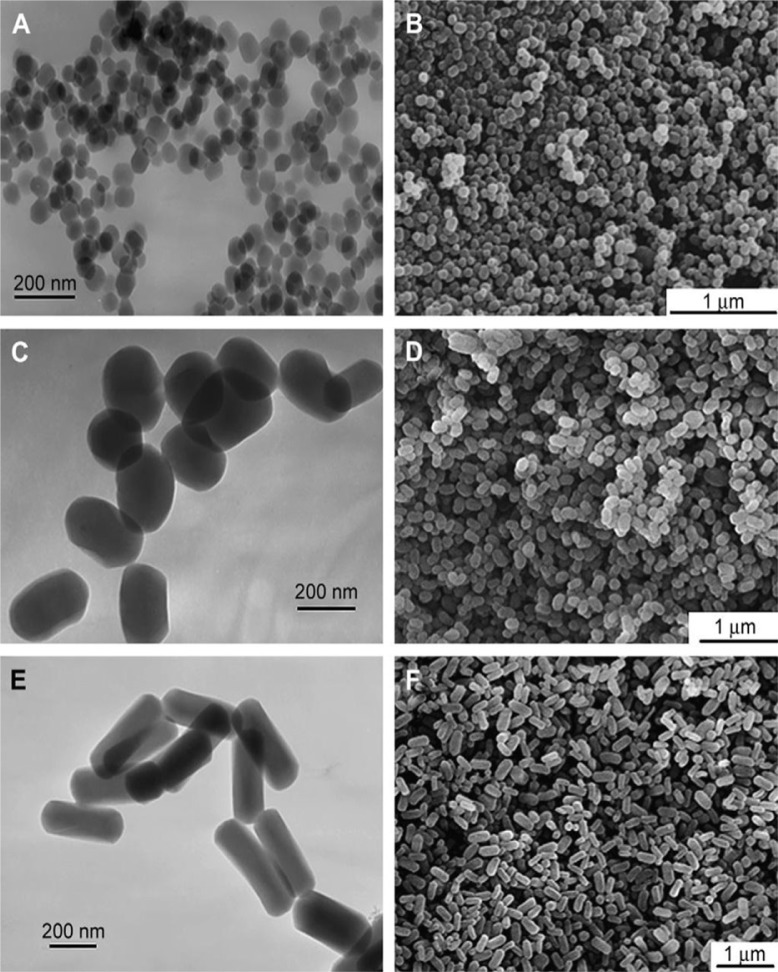
Effect of different shape of the nanoparticles on their arrangement. The TEM and SEM pictures of mesoporous silica nanoparticles in different shapes are illustrated. (A, C, E) TEM images of sphere-shaped particles (A), short rod-shaped particles (C) and long rod-shaped particles (E). (B, D, F) SEM images of sphere-shaped particles (B), short rod-shaped particles (D) and long rod-shaped particles (F) (39).

Shape is also a known factor which contributes to nanoparticle toxicity. Nanoparticles, in general, can be categorised into two groups of high aspect ratio such as nanowires and nanotubes and low aspect ratio such as nanospheres and nanocubes (11). In general, the fibre-like (large aspect ratio) or clustered nano-particles are known to be more toxic and harmful to cells in comparison to round and ringed nanoparticles (Fig. 7). It has been suggested that unlike spherical nanoparticles, the large contact area of long nanoparticles with cell surface receptors, puts a strain on the cytoskeleton of the phagocyte during phagocytosis, impeding the process (10). This was demonstrated in an experiment where nickel, in the shape of either a dendritic cluster or a sphere, was added to zebrafish embryos (46, 47), and the former showed significant toxicity in comparison to the spherical nanoparticles (10). Similarly, other groups have shown that nanoparticles with a rod structure such as silver nanorods have a greater toxicity to their spherical counterparts (48). As mentioned above, materials with a larger aspect ratio tend to be more toxic than those with a smaller one. This was demonstrated by a study which designed three monodisperese mesoporous silica (MSNs) with similar particle diameter, chemical composition, and surface charge but with different aspect ratios (ARs: 1, 2, 4) were specially designed. Each was added into human melanoma cell line (A375). The results indicated an increase of major cellular interruption by increasing the aspect ratio of nanoparticles (11, 39). In addition to the cytotoxicity of fibrous nanoparticles, their presence in the blood circulation can also cause adverse effects. In one experiment, fullerenes, SWCNT, and MWCNT were injected into the artery of a rat. This resulted in the formation of vascular thrombosis and platelet aggregation in the case of SWCNT and MWCNT but not in the case of fullerenes. It has been suggested that the fibre shape of the CNT results in stronger adherence to the blood vessel wall in comparison to spherical nanoparticles (10).

### Size of nanoparticles

**Fig. 8:**
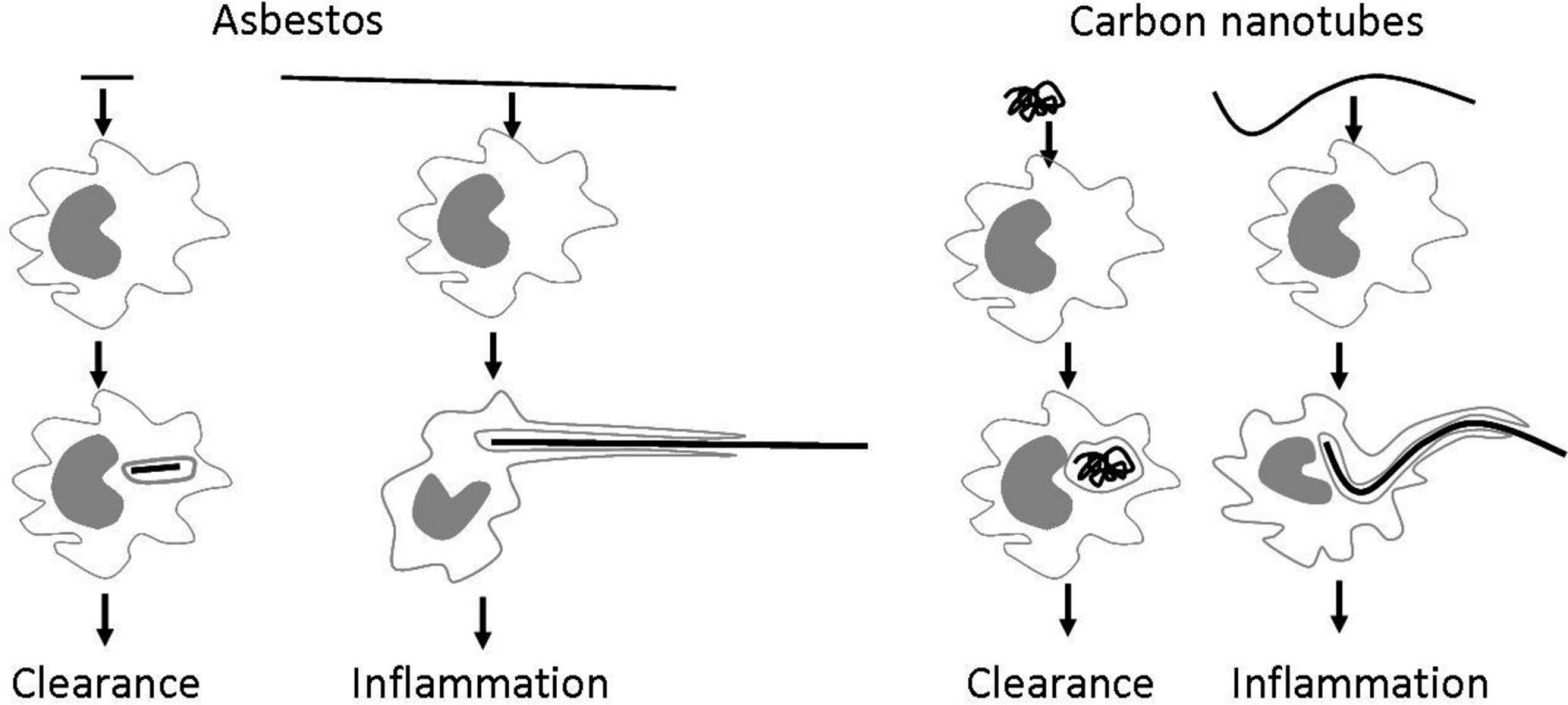
‘Small’ particles, generally regarded as shorter than 20 µm can be engulfed by macrophages and cleared. However long, fibre-like particles such as asbestos fibres or CNTs cannot be fully engulfed by macrophages, leading to frustrated phagocytosis and chronic inflammation (49).

CNT length and diameter, which can be altered during CNT synthesis is another major factor that determines its toxicity. Long fibres (>20 µm) including CNTs which exceed the length of a macrophage cannot always be fully engulfed by a macrophage leading to ‘frustrated’ phagocytosis (Fig. 8), preventing their clearance from the system and causing the release of inflammatory factors.

Various studies have shown that CNTs with longer length and larger diameter have greater toxicity than smaller ones. Another group have also shown that small-length CNTs do not result in damage to the cells. Muller et al. used both functionalised and non-functionalised short CNT (1 µm) in an *in vivo* model (rat) study. The results showed no risk of mesothelioma in both cases of functionalised and non-functionalised CNT after 24 months (50). In another experiment, researchers used the comet assay to examine the effect of CNT size on DNA damage in human aleveolar carcinoma epithelial cells (A549). In the experiment, A549 cells were cultured in a six-well plate for 24 h. Semi-confluent culture was exposed for 3 h with 50 µg/ml of MWCNT (M1, M2, and M3) with various lengths and diameters: M1 (length: 5–15 µm, diameter: 20–60 nm), M2 (length: 1–2 µm, diameter: 60–100 nm), and M3 (length: 1–2 µm, diameter: <10 nm). In addition, the extent of DNA damage caused by SWCNT (length 5–15 µm, diameter: <2 nm) was also investigated. They found that M1 caused a significant amount of DNA damage, M2 a slight amount and M3 and the SWCNTs a minimal amount (51). Despite the fact that most studies have shown that longer and wider CNTs lead to greater toxicity, some researchers have found the opposite. In one study, MWCNTs were processed in 2.6 M nitric acid for 24 and 48 h to produce lengths of 0.8±0.5 µm and 0.2±0.1 µm, respectively. Treatment of zebrafish embryos with the shorter MWCNT caused severe developmental defects, whilst no detectable defects were evident after treatment with the longer MWCNT (52). In another experiment, C6 rat glioma cell were treated with either MWCNT1 (length: 2 µm, diameter: 10–20 nm) or MWCNT2 (length: 10 µm, diameter: 40–100 nm) at various concentrations. Using the MTT assay and flow cytometry, the study found that the shorter, MWCNT1 caused increased apoptosis and G1 cell cycle arrest compared to MWCNT2 (53).

## *In vitro* and *in vivo* toxicology analysis

Using cell-culture-based assays of toxicity is a popular technique for investigating the toxicity of nanoparticles (54). Different research groups use different techniques, such as the MTT assay, the use of Alamar Blue and Trypan blue to establish the toxicity of the CNTs in *in vitro* studies. Such conventional toxicity assays are widely used and have yielded interesting results that have provided a valuable insight into nanotoxicity. However, as alluded to earlier, conflicting results on a particular nanoparticle's toxicity have been produced by different research groups. Some have suggested that this is due to the use of conventional *in vitro* toxicity assays, which are not the most appropriate assays for measuring nanoparticle toxicity and are therefore of limited use (6). For example, CNTs may interact with the dyes used in some assays, altering the colour emitted and therefore giving a false reading (37). For example, whilst most toxicology studies have shown that COOH functionalisation of SWCNT removes/reduces toxicity, Jos et al. found that COOH–SWCNT was toxic to the Caco-2 cell line (4). Another limitation of *in vitro* assays is that they cannot produce any toxicokinetic data (10). Although *in vivo* tests are more expensive and complicated, it is generally accepted that they provide more accurate and relevant information that cannot be obtained through *in vitro* studies. One example is given by the study that found that MWCNT possess teratogenic properties, as mentioned above (24). *In vivo* experiments also have the advantage that they can provide data on a wider range of parameters, such as distribution, metabolism, and elimination.

## How CNT may damage the cell during its exposure?

In general, researchers have classified the cell death programmes into three main groups: apoptosis, autophagy, and necrosis. Apoptosis is programmed cell death and can be activated via the extrinsic (death-receptor-dependent) or intrinsic (mitochondrial-dependent) pathway and it may or may not require the activation of cytosolic proteases known as caspases (55). Autophagy involves the cell essentially eating itself and necrosis a less tightly controlled, pathological, and chaotic form of cell death. It is at present unclear, which a cell death programme is induced in response to exposure to different types of CNTs. In the previously mentioned study of MWCNT's effects on A549 lung cells, the authors indicate that the cells may have undergone apoptotic cell death.

Various experiments have shown that lysosomal damage is one of the main reasons for CNTs to trigger apoptosis. It has been suggested that the exposure of CNT to the cell's environment results in destabilisation of lysosomal membranes leading to apoptotic as well as necrotic cell death. Mitochondrial damage, which eventually leads to lysosomal damage, is also another way, which has been suggested by other research groups, as a method for the induction of apoptosis by the CNT exposure to the cell. The injured lysosome releases digestive enzymes, which damage entire cells (55). It has been suggested that the CNT could result in a decrease of mitochondrial membrane potential (MMP), which will result in the formation of reactive oxygen species (ROS), an increase in the level of lipid peroxide and decreased activities of superoxide dismutase (SOD), glutathione peroxidase (GSH-Px), catalase (CAT), and the content of glutathione (GSH) in a dose-dependent manner (5). A high level of ROS is indicative of oxidative stress and can damage cells by altering protein structure, disrupting DNA, interfering with signalling functions, and modulating gene transcription. This can eventually result in cancer, renal disease, neurodegeneration, cardiovascular or pulmonary disease. The effect of high level of ROS is even more detectable in the central nervous system due to the high content of unsaturated fatty acids, which are susceptible to peroxidation (10). In one experiment, rat lung epithelial cells (LE) were treated with varying concentrations (0.5–10 µg/ml) of MWCNT, resulting in the induction of apoptosis through mitochondrial damage (56). In another study, it was found that the incubation of monocytic cell line RAW 267.4 cells with MWCNT resulted in significant suppression of differentiation into osteoclasts. The results also indicated that the treatment of MWCNT induced apoptosis as characterised by nuclear condensation, DNA fragmentation, caspase-3 activation, poly (ADP-ribose) polymerase (PARP) cleavage and release of cytochrome c from the mitochondria to the cytosol (57). A different experiment conducted by another group on the same cell line (RAW267.4) has also determined that MWCNT functionalised with carboxylic acid would also result in apoptosis through the damage to the mitochondria (9). The other methods that nanoparticles would result in cell's interruption are its effect on Golgi apparatus and damage to the DNA's function (58). As well as apoptosis process, it has also been suggested that the CNT's exposure can also lead into the autophagy activations. Despite the fact that functionalisation has been reported as a method for reducing the CNT toxicity, –COOH-functionalised SWCNTs were found to induce autophagy in lung adenocarcinoma A549 cells, whilst the addition of SWCNT–PABS (polyaminobenzene sulfonic acid) and SWCNT–PEG had no such effect (59).

## Conclusion

CNTs have enormous potential for clinical application; however, this will only be realised once they can be made safe and their toxicity is fully understood. Studies so far have taught us that factors determining CNT toxicity include length, diameter, purity, production method and functionalisation, and that by modifying these factors, CNTs may be safe for human use. *In vivo* studies have been informative in demonstrating that different methods of administration result in different pathologies. Whilst *in vitro* studies have been useful in identifying the determinants of CNT toxicity, drawing clear conclusions from the literature is sometimes made difficult by the inconsistency between studies. This perhaps reflects the growing opinion that conventional cytotoxic assays are not suitable for nanotoxicity experiments because nanoparticles have unique properties and therefore may alter the outcome of an experiment in unpredictable ways. This presents a need for greater standardisation in the field and a consensus on appropriate ways to measure nanotoxicity. Nevertheless, based on our findings as well as others, specific types of functionalisation can greatly minimise CNT toxicity and represent promising progress towards their clinical use.
